# How to Create Memorizable and Strong Passwords

**DOI:** 10.2196/jmir.1906

**Published:** 2012-01-10

**Authors:** Pietro Cipresso, Andrea Gaggioli, Silvia Serino, Sergio Cipresso, Giuseppe Riva

**Affiliations:** ^1^Applied Technology for Neuro-Psychology LabIRCCS Istituto Auxologico ItalianoMilanoItaly; ^2^Psychology DepartmentCatholic University of MilanMilanoItaly; ^3^FreelancerMilanItaly

**Keywords:** Privacy, security, passwords, psychology

## How to Create Memorizable and Strong Passwords

In a recent JMIR article, El Emam, Moreau and Jonker highlight the importance of using strong passwords to protect personal health information in clinical trials [1]. An important implication that was not fully discussed is the potential problem people may have to create passwords that are complex but at the same time easy to remember.

**Figure 1:**
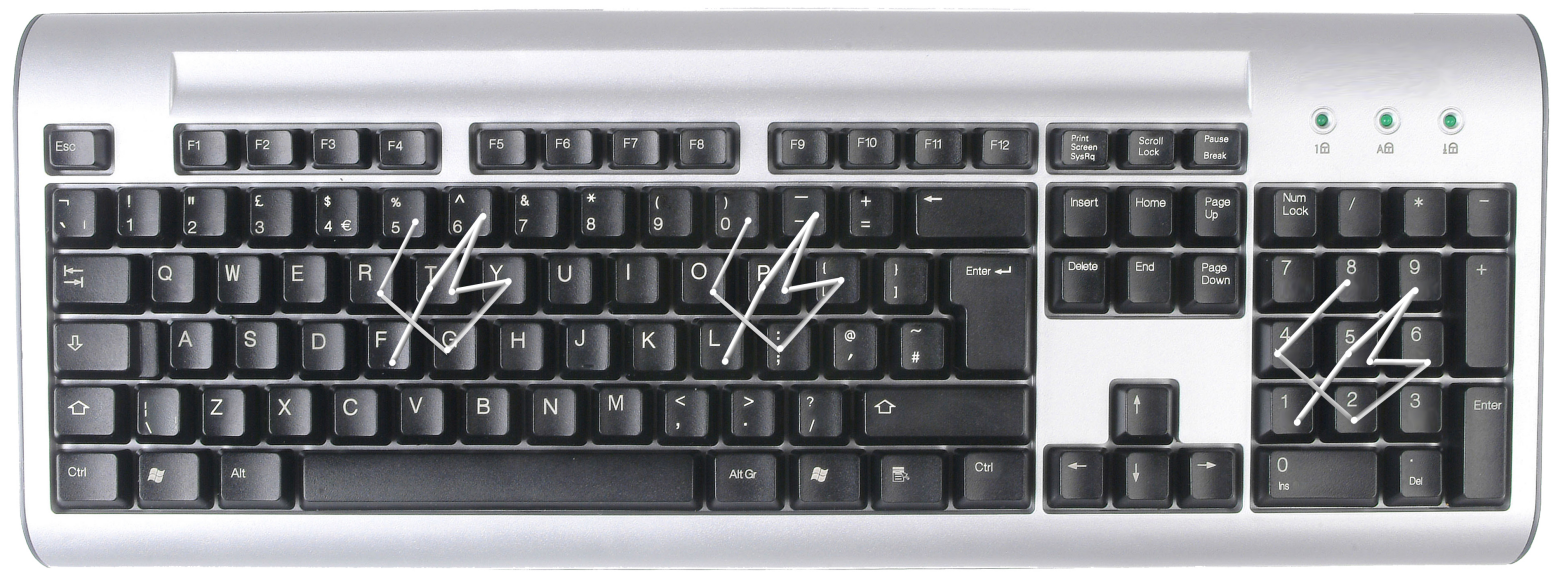
The same sequence to generate different passwords (about 15 minutes to memorize the sequence).

**Figure 2:**
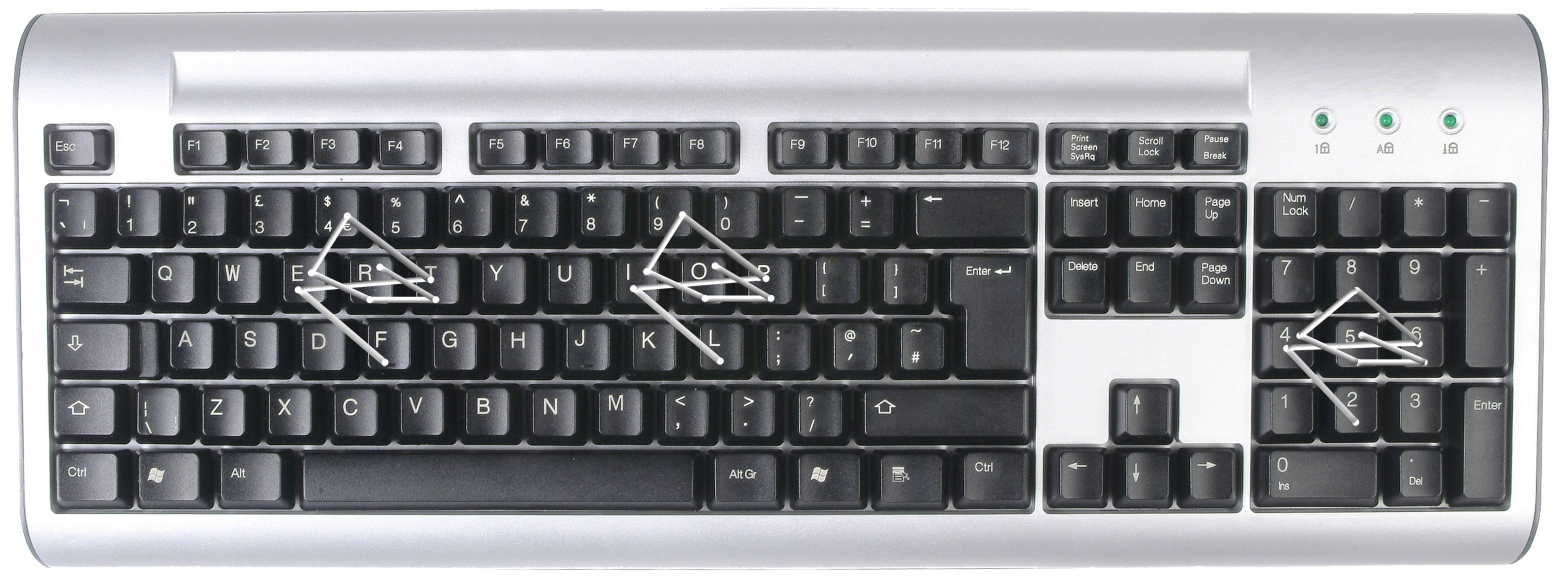
Another sequence to generate other passwords (about 15 minutes to memorize the sequence).

To address this problem we propose the PsychoPass methord, a simple way to create strong passwords which are easy to remember. This method relies on mental practice and is not an hardware or a software to download. The idea is that a password can be created, memorized and recalled by just thinking of an *action sequence* instead of a word or string of characters. To be more specific, the method consists of the following steps (see Figure 1 and 2): (1) begin with a letter on the keyboard; (2) memorize a sequence of actions (something like “the key on the left, then the upper one, then the one on the right”, and so on); (3) memorize the sequence (not the letters used); (4) create as many passwords as you want by remembering only the first letter and the sequence. Using different types of sequences it is possible generate thousands of different passwords. Using sequences' combination is possible to create an infinite number of passwords. Moreover the created passwords will be a nonsense sequence of letters, numbers and symbols, resilient to any attack.

Furthermore the password communication among colleagues maybe done just by using the first letter and on the base of a common knowledge of the sequence (e.g., sequence 3, letter j).

El Emam and Colleagues state that more sophisticated collaboration tools are required to allow file sharing without password sharing, and provide several recommendations to implement these practices. We think that more awareness and new practices among users may represent the correct way to implement security beyond the technological issues. In particular, future research needs to focus on the processes that make technology a powerful tool for security.

## Multimedia Appendix 1

Movie demonstrating the method.
